# Supplementary material for: Spatial Variation in Stream-Water Bacterial Communities Across Sites Differing in Wild Chinese Giant Salamander Detection Frequency
Source: Biology (Basel). 2026 Sep 18;15(18):1647. doi: 10.3390/biology15181647 (PMC13604818; doi:10.3390/biology15181647)
Supplement: Supplementary file 1 [file biology-15-01647-s001.zip › Data Related to “Spatial Variation in Stream-Water Bacterial Communities across Sites Differing in Wild Chinese Giant Salamander Detection Frequency”/Microbial Data/result/08.FunctionPrediction/picrust2/pca/sample/TIGRFAM/PCA_circleLable.pdf]

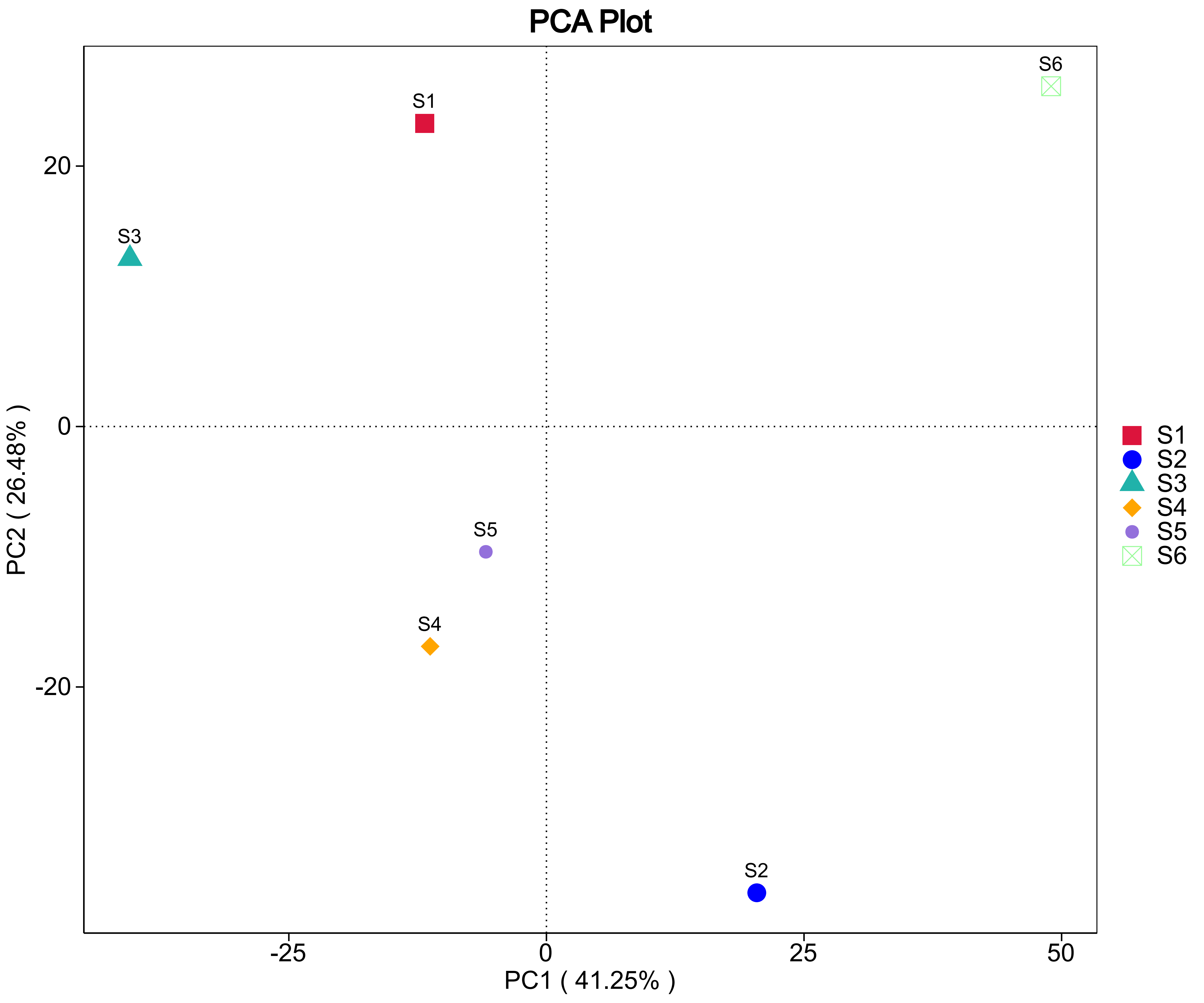
PCA Plot
S1
S2
S3
S4
S5
S6
20
0
-20
-25
0
25
50
PC1 ( 41.25% )
PC2 ( 26.48% )
S1
S2
S3
S4
S5
S6
